# Influence of the Times upon the Dental Profession

**Published:** 1862-11

**Authors:** J. Taft


DENTAL REGISTER OF THE WEST.

VOL. XVI.] NOVEMBER, 1862. [NO. 11.

### Original Essays and Communications.

### INFLUENCE OF THE TIMES UPON THE DENTAL PROFESSION.

BY J. TAFT.

IN all departments of occupation and industry, there are great changes and modifications, induced by the disturbed condition of affairs in our country; there is a breaking up of the fountains of the deep, an overturning of usages and customs. Amid all this, the dental profession will necessarily be much influenced. How far it may arrest the progress and development may not at once be apparent, nor indeed is it certain that it will be at all checked, except it may be, perhaps, through a temporary diversion of the mind to other subjects. This, of course, to a greater or less extent has been done; but this may prove a mere relaxation, from which the mind will soon revert with renewed energy to its wonted work. Some are disposed to lament the fact that there is now so much outside of our specialty to occupy the mind that it is liable to be wholly absorbed, independent of professional matters. But he must be very dull indeed who does not know that the people have far more activity of mind—think far more than they have been accustomed to do, when all was peace and quiet. This is apparent from many considerations; a very great number of diverse subjects are rapidly presented to the mind for consideration, and again, the mind of the people was never better attuned for vigorous thinking than at the present. The health of our people has been vastly better for the last year and a half than ever before ; we except, of course, diseases and affections incident to army life.

Great and momentous ideas and questions are presented to be considered and decided upon immediately ; if they remain a few hours, or a day or two, they become stale, and their import and interest are most probably gone ; they are superseded by others, that demand as rapid consideration and as hasty decision.

These circumstances, then, cultivate vigorous, rapid thinking and prompt decisions ; this habit will not be confined to the great events that have been the occasion of it, but it will influence every department of life. In this, then, we think there is ample compensation for any temporary mental diversion that may have occurred.

Another result is, the withdrawal of a large number of operators from the ranks of the profession. This was brought about by two or three causes ; dentists, in common with other people, are patriotic ; and again, upon the first outbreak, the dental practice, in common with other business, was very much affected ; indeed, in a great many cases was so much reduced, that practitioners gave up their business ; some went into other occupations, but for the most part they entered the army. With very few exceptions, those who were thus thrown out of the profession were not those who did the most for the progress and development of the profession ; the real active, earnest workers are yet in the profession, and enjoying its emoluments as bountifully, perhaps, as ever, and in a great many instances, we think, more so ; for the panic having at last subsided, the people find their teeth continue to decay, and that their requests for the service of a dentist are as great as ever, and money is more plenty than ever, and more universally diffused, so that a greater number now have the ability to avail themselves of the services of the dentist than ever before, and the number of operators is at least twenty-five per cent. less.

The removal of an unhealthy competition, and increased patronage, will enable the present operators to improve and perfect their operations, and be remunerated for them; and the circumstances should be improved to that end. We advise this because it is to the advantage of all concerned; it promotes the profession, does good in many ways to the dentist, and would be of incalculable value to the patient.

We can not conclude, then, that the present state of affairs is prejudicial to the progress of our profession.

We have an earnest of what will continue to be done, in the fact that we had two of the best national dental meetings, within the last three months, that it has ever been our privilege to attend.

